# The "New Hospitals."

**Published:** 1920-05-29

**Authors:** 


### THE "NEW HOSPITALS."

The provisions of the Health Services Bill, especially those relating to the hospitals on the Bradford model, or what are known as the "New Hospitals," and also as to how the new hospital will affect the old hospital, are looked forward to with much interest. There is little doubt that the provisions of the Bill will be to a large extent in accordance with the recommendations of the Consultative Council on Medical and Allied Services, whose report will be found on another page; but, as we stated some weeks ago, Dr. Addison is not likely in his Bill to tie his hands with too much detail. He will probably suggest a framework for a scheme of health service, and will take comprehensive powers for future use in filling in the blank panels. His scheme will surely be one which may include as the model for the "New Hospitals" the Primary Health Centres and Secondary Health Centres suggested by the Consultative Council (see p. 221).

The fact that there is a great deal of hospital accommodation in insufficient or uneconomical use at the present time cannot have been overlooked by the Health Ministry. In the Metropolis the provision of beds made by the voluntary hospitals for each thousand of the population is 1.825; that made by the Poor Law is 2.450; by the fever hospitals, 1.182; and by convalescent homes, .042. Very possibly the number of beds required for an ideal system has not yet been reached, but the important point arises as to whether we are making the best use of the beds we already have.

Lord Knutsford, at a recent meeting at the London Hospital, stated that at a time when the London Hospital had more than 800 names on its waiting-list there were at the infirmary across the road 200 empty beds. There was nothing to prevent these beds being used except Poor-Law regulations. Mr. Morris, of the same great hospital, in his well-remembered *Daily Telegraph* article, vividly described the disadvantage of the want of a working arrangement between hospital and infirmary, advocating the hospital, with its quick turnover, almost as a clearing station, the infirmary for the longer-staying cases, and the convalescent home for the finishing touches. The advantage to the medical student and the nurse in training is manifest, as illnesses of all kinds could be studied and nursed at all stages up to convalescence. As we stand, the hospital, if it wishes to make any impression on the list of applicants, has to be cruel in detail and kind in the gross, by discharging patients, long before any approach to convalescence is reached, for continued treatment and such attention as is possible in their own homes. Since the article was written St. Mary's has made its arrangement with Paddington Infirmary under which the hospital supplies a consulting staff, who visit the infirmary regularly and look after such services as pathology and bacteriology, the students being freely admitted to the wards.

It will appear to many that in a large number of cases the Poor-Law Infirmary, re-named, improved by special departments, with the medical staffs augmented, might very well in some instances become the "New Hospitals." The buildings in many cases may be described not merely as palatial, but, what is better, perfectly suited in essentials for the treatment of much larger numbers of curable illness. Dr. Addison knows this, and we have little doubt that the Health Services Bill will provide for the better and fuller use of infirmaries. It is probable that if the infirmaries kept their beds full, and were in a position to undertake surgical operations on the scale general in other hospitals, the problem of accommodation would be solved without embarking on ambitious building operations. The solution does not involve capital expenditure; further, it makes no large call for labour upon the building trades.

We notice with pleasure that the Consultative Council recommends that facilities shall be given to the general practitioner to follow his case through the whole of the treatment, and, indeed, in the Primary Health Centre to have the patient under his own care. In the Secondary Health Centre also the patient, if it is desired, and his financial position allows him to do so, may choose his own specialist. The medical profession, very rightly, at the passing of the National Health Act held out for free choice of doctor by the patient and free choice of patient by the doctor. It is very satisfactory that this principle underlies the scheme suggested by the Consultative Council.

